# Survey of selected tick-borne diseases in dogs in Finland

**DOI:** 10.1186/1756-3305-7-285

**Published:** 2014-06-23

**Authors:** Cristina Pérez Vera, Suvi Kapiainen, Sami Junnikkala, Kirsi Aaltonen, Thomas Spillmann, Olli Vapalahti

**Affiliations:** 1 Haartman Institute, Department of Virology, University of Helsinki, P.O. Box 21, 00014 Helsinki, Finland; 2 Department of Equine and Small Animal Medicine, University of Helsinki, PO 57 (Viikintie 49), 00014 Helsinki, Finland; 3 Department of Clinical Veterinary Studies, Vetsuisse Faculty, University of Bern, Länggassstrasse 128, 3012 Bern, Switzerland; 4 Department of Veterinary Biosciences, University of Helsinki, P.O. Box 66, 0014 Helsinki, Finland; 5 Department of Veterinary Biosciences, Division of Microbiology and Epidemiology, University of Helsinki, P.O. Box 66 (Agnes Sjöbergin katu 2), 00014 Helsinki, Finland; 6 Department of Virology, Helsinki University Central Hospital Laboratory, P.O. Box 400 (Haartmaninkatu 3), 00029 Helsinki, Finland

**Keywords:** Anaplasmosis, Borreliosis, Bartonella, Dogs, Epidemiology

## Abstract

**Background:**

Due to climate changes during the last decades, ticks have progressively spread into higher latitudes in northern Europe. Although some tick borne diseases are known to be endemic in Finland, to date there is limited information with regard to the prevalence of these infections in companion animals. We determined the antibody and DNA prevalence of the following organisms in randomly selected client-owned and clinically healthy hunting dogs living in Finland: *Ehrlichia canis* (Ec), *Anaplasma phagocytophilum* (Ap), *Borrelia burgdorferi* (Bb) and *Bartonella*.

**Methods:**

Anti-Ap, −Bb and –Ec antibodies were determined in 340 Finnish pet dogs and 50 healthy hunting dogs using the 4DX Snap®Test (IDEXX Laboratories). In addition, PCRs for the detection of Ap and *Bartonella* DNA were performed. Univariate and multivariate logistic regression analyses were used to identify risk factors associated with seropositivity to a vector borne agent.

**Results:**

The overall seroprevalence was highest for Ap (5.3%), followed by Bb (2.9%), and Ec (0.3%). Seropositivities to Ap and Bb were significantly higher in the Åland Islands (*p* <0.001), with prevalence of Ap and Bb antibodies of 45 and 20%, respectively. In healthy hunting dogs, seropositivity rates of 4% (2/50) and 2% (1/50) were recorded for Ap and Bb, respectively. One client-owned dog and one hunting dog, both healthy, were infected with Ap as determined by PCR, while being seronegative. For *Bartonella* spp., none of the dogs tested was positive by PCR.

**Conclusions:**

This study represents the first data of seroprevalence to tick borne diseases in the Finnish dog population. Our results indicate that dogs in Finland are exposed to vector borne diseases, with Ap being the most seroprevalent of the diseases tested, followed by Bb. Almost 50% of dogs living in Åland Islands were Ap seropositive. This finding suggests the possibility of a high incidence of Ap infection in humans in this region. Knowing the distribution of seroprevalence in dogs may help predict the pattern of a tick borne disease and may aid in diagnostic and prevention efforts.

## Background

Vector borne diseases are increasingly recognized as the cause of several clinical illnesses in humans and domestic animals. These include infections transmitted by fleas and ticks, as well as other hematophagous arthropods. For several reasons, their epidemiology in Europe is changing [1,2]. In the recent literature, there are several case reports of tick borne diseases diagnosed in previously non-endemic areas, both in human and veterinary medicine. Ticks and the diseases they transmit generally have a zoogeographical range determined by host movement and climatic factors. Variations in temperature and humidity, especially global warming, affect the arthropod abundance, distribution and vector capacity. The most common tick in North-Western Europe is the sheep or castor bean tick, *Ixodes ricinus*, which is widely distributed in Finland [3]. *Ixodes* ticks are vectors of a broad range of pathogens of medical and veterinary importance [1], such as *Babesia* spp., *Borrelia* spp., *Anaplasma phagocytophilum* (Ap), *Bartonella* spp., tick-borne encephalitis virus (TBEV), and *Francisella tularensis*. In Finland, however, there is limited information regarding the prevalence of many vector borne diseases in companion animals, and thus the majority of available data come from human medicine studies. Many of the arthropod-borne infections that affect dogs can cause serious disease in people, and therefore dogs have often been considered to serve as effective sentinel animals to assess the risk of human infection [1].

Infections with Ap, the causative agent of human granulocytic ehrlichiosis in the US, have been increasingly diagnosed in people and dogs living in Bb-endemic areas [1,4]. Previously, Anaplasma infections, so called Tick-borne fever, were reported in cattle and sheep in Finland [5-7]. *Anaplasma phagocytophilum* usually causes an acute infection in dogs characterized by fever and thrombocytopenia, although subclinical infections have been reported [4]. Recently, Ap infection has been reported in one cat [8], two dogs [9] and one horse in Finland [10]. This pathogen was recently found in *Ixodes ricinus* ticks from the southeastern part of the country [11].

Spirochetes of the *Borrelia burgdorferi* (Bb) sensu lato complex cause Lyme disease, the most commonly reported vector-borne disease in Europe [1,12]. The prevalence of Bb infection varies geographically and follows the distribution of *Ixodes ricinus* and *Ixodes persulcatus*, the primary vectors of Bb. This disease is known to be endemic in Finland [13]. *I. persulcatus* is found along the western coast, whereas *I. ricinus* is distributed in the southern and central parts of the country [3]. To date, infection with Bb has been associated with neuroborreliosis, erythema migrans, arthritis and other musculoskeletal symptoms in humans [1]. In contrast, the majority of exposed dogs remain asymptomatic [14]. However, protein-losing nephropathy with renal failure has been associated with Lyme disease in dogs. To the authors’ knowledge, the Bb seroprevalence has not been studied in dogs in Finland and much remains unknown regarding epidemiology of canine Bb infection in the country. In addition to Bb, *I.ricinus* harbors a great diversity of organisms potentially pathogenic for humans and dogs, including *Bartonella spp.*[15]. Co-infection of *Bartonella spp.* with Bb and other known tick-borne pathogens such as Ap or *Babesia spp.* has been recognized in ticks and hosts infected with *Bartonella* spp [15].

*Ehrlichia canis* (Ec) is the causative agent of canine monocytic ehrlichiosis and is transmitted by the brown tick *Rhipicephalus sanguineus.* Three clinicopathologic stages of ehrlichiosis have been recognized in dogs [1]: an acute stage, where dogs show variable clinical signs (such as lethargy, fever, lymphadenomegaly, epistaxis) and the bloodwork reveals mostly thrombocytopenia with or without anemia; a subacute phase, characterized by hyperglobulinemia, thrombocytopenia and anemia; and a third or chronic stage, where dogs may have variable clinicopathologic findings (lethargy, thrombocytopenia, pancytopenia) and remain seropositive. To date, no studies have reported the Ec exposure rates in dogs living in a non-endemic area like Finland.

Members of the genus *Bartonella* are Gram-negative hemotropic bacteria that are transmitted by several arthropod vectors, including *Ixodes* ticks, blood transfusion, and via animal scratches and bites [16]. At least eight *Bartonella* species have been implicated as canine pathogens [17]. There appears to be a growing spectrum of arthropods that might serve as potential vectors for *Bartonella* species [15]. The deer ked, *Lipoptena cervi,* is a blood-sucking ectoparasite of moose (*Alces alces*), which has been found to harbor *Bartonella* DNA [18]. At this point it remains to be determined if the deer ked can successfully transmit *Bartonella* to moose or other mammals. The deer ked has drawn strong public attention in the last years in Finland, as this parasite has been rapidly spreading northward from the Southeast and dispersing into new areas [19]. The incidental infestation of deer keds to humans is well known in Finland, which is a nuisance for people who participate in outdoor activities, such as hunters, berry pickers, as well as other people who spend time in forested areas during late summer and early autumn. To date, no dog with *Bartonella* infection has been reported in Finland.

This cross-sectional study was designed to establish the serological (Ec, Ap, Bb) and molecular prevalence (Ap, *Bartonella*) of selected tick borne diseases in dogs in Finland, and determine the geographical distribution and epidemiological factors associated with exposure and/or infection.

## Methods

### Study population

The study protocol was approved by the Laboratory Animal Board of the Southern Finland Regional State Administrative Agency. Three hundred and forty anti-coagulated blood and serum samples from dogs living in Finland were included in the study. Of these, two hundred and nineteen samples were collected from client-owned dogs evaluated in private practices around the country and at the veterinary teaching hospital of the University of Helsinki, in the fall (September to November) of 2011 and 2012. Furthermore, one hundred and twenty-one samples from client-owned Finnish dogs, which had been collected in the fall of 2010 and 2011 and had been stored in a blood bank at −30°C, were also included in the study. Lastly, 50 healthy hunting dogs were included in the study, from which blood samples were collected during a hunting dog show in September of 2011.

The blood from pet dogs was randomly collected in the following manner: in the summer of 2011 and 2012, approximately fifty veterinary clinics from all around Finland were randomly contacted per email to inform them about our study. The contact information had been obtained via internet (http://www.fonecta.fi, key words: eläinlääkäri suomi). In less populated areas of Finland, where a small number of clinics were found (Lapland, Åland), the veterinarians were contacted by phone. Of the 50 clinics contacted, twenty-nine agreed to participate in the study, which received a pre-paid envelope with detailed instructions for blood collection/storing/shipping as well as EDTA (Ethylene diaminetetraacetic acid) and serum tubes. In order to avoid any bias selection, the veterinarians were specifically asked to collect blood from any dog presented at their clinic/hospital during one week (any, as soon as they received the envelopes) between September and October, regardless of the clinical signs of the dog.

### Data collection

For the pet dog population, the data of sample collection, age, breed, size (defined as small ≤10 kg, medium 11–25 kg, and large >25 kg), sex and neuter status, as well as municipality and zip code, were documented. Whether the animal had a travel history outside Finland was also recorded. Finally, it was also documented whether the animal showed any clinical signs of illness at the time of blood collection.

For the healthy hunting dog group, date of collection, age, sex as well as municipality and zip code were recorded.

For statistical purposes, municipalities and zip codes were categorized into 6 regions (historical provinces) in Finland: Lapland, Oulu, Eastern Finland, Western Finland, Southern Finland and Åland.

### Serological testing

Serum samples were tested for the presence of Ec*,* Ap and Bb antibodies using a qualitative dot-ELISA SNAP 4DX ® (IDEXX Laboratories).

### DNA extraction and PCR amplification

DNA was extracted from 300 μl of each dog’s frozen EDTA-blood pellet using a commercially available GFX Genomic Blood DNA Purification Kit (Qiagen, Germany). The final eluted volume was 200 μl per sample.

PCR screening for *Bartonella* DNA was performed targeting the intergenic spacer (between 16S sRNA and 23S rRNA region) using primers (BsppITS325s: 5′ CCTCMGATGATGATCCCAAGCCTTYTGGCG 3′ and BsppITS1100as: 5′-GAACCGACGACCCCCTGCTTGCAAAGCA-3′) as described previously [20]. Amplification was performed in a 25 μL final volume reaction containing 12,5 μl of the Phusion Flash master mix (Fisher Scientific, USA), 200 nM of each primer and 5 μL of DNA template. For the detection of *Anaplasma* DNA, a quantitative PCR based upon amplification of the multicopy *msp2* gene was performed, modified from a previously described protocol [21]. Briefly, the reaction was performed at 20 μl final volume containing 10 μl of PerfeCta qPCR ToughMix 2x (Quanta BioSciences, USA), 750 nM of the forward primer 5′-GAAGATGAWGCTGATACAGTA-3′, 750 nM of the reverse primer 5′- CAACHGCCTTAGCAAACT-3′, 200 nM of the probe Fam- TTATCAGTCTGTCCAGTAACA -Tamra and 5 μl of template DNA. The Stratagene MX3005P thermocycler was used to run the program with an initial denaturation step of 1 min at 95°C followed by 50 cycles of 10 s at 95°C, 10 s annealing at 53°C and 8 s extension and measurement at 72°C.

### Statistical analysis

Logistic regression analysis was carried out in order to assess associations between each factor (sex age-group, size-group, geographic region, travel history and health status) and the prevalence to each of the arthropod borne diseases studied. Each factor was first analyzed separately with univariate logistic regression. Each model included only the factor at hand as fixed effect. A liberal alpha value was selected (*p* ≤ 0.1) as an entry criterion for exact logistic regression analysis. Variables that were significant at the univariate analysis were subsequently individually entered into a multivariable logistic regression analysis, for which significance was set at *p* ≤ 0.05. With the multivariable model the possible correlations of the factors could be taken into account. In the modeling the differences between groups were quantified with odds ratios (OR) and their 95% confidence intervals (CI). All the models were constructed to model the risk of having a tick borne disease. Statistical analyses were performed using 4Pharma Ltd using SAS® System for Windows, version 9.3 (SAS Institute Inc., Cary, NC, USA).

## Results

**Figure 1:**
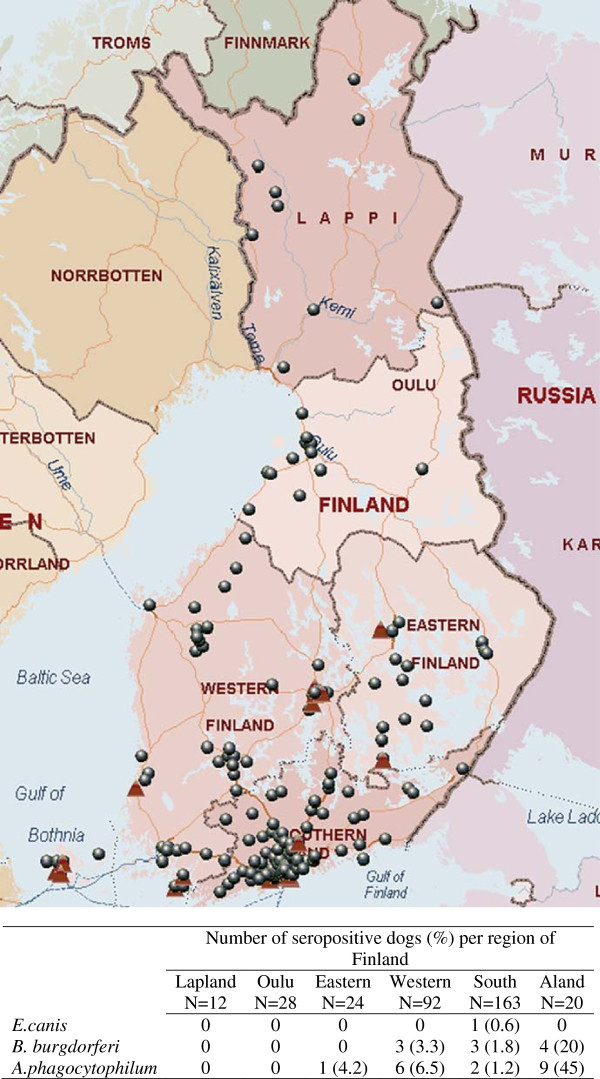
**Distribution of the 193 zip codes recorded from 340 pet dogs included in our study.** The dots denote the dog samples that were seronegative to all vector borne diseases tested, whereas the triangles show the samples that were seropositive to at least one infectious disease. The seroprevalence for every vector borne disease in each region in reported in the table.

Three hundred and forty client-owned pet dogs as well as 50 healthy hunting dogs were included in the study. Dog samples were submitted from veterinary clinics and hospitals from South (163 dogs, 10 clinics), Western (92 dogs, 6 clinics) and Eastern Finland (24, 6 clinics), Oulu (28, 2 clinics), Åland (20, 2 clinics) and Lapland (12, 3 clinics). A total of 193 zip codes were recorded (Figure 1). Altogether 98 different dog breeds were reported. Travel history was available in 193 dogs, of which 27 had a history of being abroad (Germany, Poland, Sweden, Denmark, Norway, Estonia, France, Spain, USA, Italy and Latvia). Other demographic information recorded from pet dogs is available in Table 1.

**Table 1:** **Association between signalment, size, origin, health status and travel history between dogs that were seropositive and seronegative to any of the tick borne diseases tested**^**a**^

**Characteristic**	**Categories**	**Seropositive (N = 29)**	**Seronegative (N = 311)**	**Univariate p-value**^**b**^
		**N (%)**	**N (%)**	
**Origin**	South	0	156 (96.3)	**<0.001**
	East	1 (4)	24 (96)	
	West	10 (10.9)	82 (89.1)	
	Oulu	0	28 (100)	
	Lapland	0	12 (100)	
	Åland	12 (60)	8 (40)	
	Missing data		1	
**Age**	≤2 years	6 (5.6)	101 (94.4)	0.49
	2-8 years	18 (9.6)	170 (90.4)	
	>8 years	4 (9.1)	40 (90.9)	
	Missing data		1	
**Sex**	Male Intact	7 (5.1)	130 (94.9)	0.3896
	Male castrated	2 (12.5)	14 (87.5	
	Female intact	12 (7.9)	139 (92.1)	
	Female spayed	3 (14.3)	18 (85.7)	
	Missing data	5	10	
**Size**	<10 kg	10 (15.2)	56 (84.8)	0.0747
	10-25 kg	8 (9.6)	75 (90.4)	
	>25 kg	10 (5.7)	165 (94.3)	
	Missing data	1	15	
**Travel history abroad**	Yes	4 (14.8)	23 (85.2)	0.6179
	No	19 (11.4)	147 (88.6)	
	Missing data		141	
**Health status**	Healthy	28 (10.0)	251 (90)	0.0652
	Sick	1 (1.6)	60 (98.4)	
	Missing data	19 (40.4)	51 (54.8)	

^a^All data presented as number of dogs (%). ^b^Univariate analysis performed with Fisher’s exact test. Results statistically significant are highlighted in bold.

*Anaplasma phagocytophilum* antibodies were detected in 5.3% (18/340) client-owned Finnish dogs in this study. Ten (2.94%) and one (0.3%) dog, which had not been abroad, were seroreactive to Bb and Ec antigens, respectively. Seropositivity to Ap and Bb was significantly higher in Åland Island, where the seropositivity rate was 45 and 20%, respectively. The seroprevalences per region are available in Figure 1. One client-owned dog, reported to be healthy, was infected with Ap, as determined by PCR, but was seronegative to Ap antigens, suggesting an acute infection. In addition, one dog from Åland was found to be both Ap and Bb seropositive. Based on logistic regression analysis, there was no significant difference in age, breed, sex, health status and travel history between seropositive and seronegative dogs (Table 1); however, living in Åland [OR = 26.65; 95% CI: 9.58-74.12] was strongly associated with an increased likelihood of being seropositive for a vector borne disease. Odds ratios, 95% confidence intervals, and *p* values obtained from the exact computations are presented in Table 2.

**Table 2:** Results of the multivariate logistic regression analysis

** Comparison**	**Odds ratio (OR)**	**Standard error**	**95% CI**		***p *value**
			**Lower**	**Upper**	
**Healthy vs Sick**	3.176	1.065	0.391	25.792	0.279
**Small vs medium**	1.392	0.614	0.416	4.660	0.591
**Small vs large**	2.710	0.575	0.874	8.409	0.084
**Medium vs large**	1.948	0.569	0.636	5.963	0.242
**East vs South**	1.012	1.113	0.113	9.041	0.992
**West vs South**	3.106	0.552	1.048	9.205	0.041
**West vs East**	3.070	1.084	0.364	25.901	0.302
**Åland vs South**	55.212	0.743	12.802	238.122	**<0.001**
**Åland vs East**	54.565	1.187	5.284	563.502	**<0.001**
**Åland vs West**	17.755	0.693	4.544	69.525	**<0.001**
**Åland vs rest of Finland**	26.647	0.52	9.580	74.118	**<0.001**

Results statistically significant are highlighted in bold.

Our study also included 50 healthy hunting dogs living in Southern Finland, which were not included in the statistical analysis, as not all the demographic information was available for all of them, and a very small number of dogs (3) were seropositive. Most of the dogs were Finnish hounds (34). Other breeds included Swedish elkhound (4), Labrador Retriever (3), German Shorthaired Pointer (2), English Springer Spaniel (2), German Hunting Terrier (2), West Siberian Laika (1), Finnish Spitz (1) and working Jack Russell Terrier (1). Eighteen were female and 32 were males, but the neuter status was not reported. Within the clinically healthy hunting dogs group, 4% (2/50) and 2% (1/50) had detectable Ap and Bb antibodies, respectively. Like in the pet dog population, one dog was infected with Ap but seronegative to Ap antigens.

For *Bartonella* spp., none of the dogs tested (pet and hunting dogs) was positive by PCR.

## Discussion

This is the first study to investigate exposure to multiple tick borne pathogens in domestic and hunting dogs in Finland. Previous studies have been limited to case reports [8-10]. The serological results obtained indicate that dogs in Finland are exposed to at least one of four vector-borne pathogens, including Ap, Ec and Bb. Our pet dog population was most frequently exposed to Ap, followed by Bb. We examined selected epidemiologic variables to assess possible associations with seropositivity to a vector borne disease. No link was detected between sex, age, travel history and health status, however, small dogs were more likely to be Ap seropositive (*p* = 0.07) compared to dogs with a weight >10 Kg. Uni- and multivariate analyses found a strong association between a particular geographic region (Åland) and being seropositive to Ap or Bb. The Åland Islands in Finland, with a population of 28,000, are known to be endemic for tick borne diseases [22,23]. Åland is an archipelago that includes Main Åland, which is group of larger islands, and more than 6,000 smaller islands. The incidence of Bb infection in people on Åland is reported to be 50 times higher than in main Finland (Finland’s National Institute for Health and Welfare, [24]). An epidemiologic study conducted in the islands showed that 85% of the people in Åland had been bitten by ticks [23]. However, to the authors’ knowledge, no published data concerning human granulocytic anaplasmosis in the Åland Islands exist. Our results suggest that Ap infections, in addition to Lyme disease, may be endemic in this region. Future studies are necessary to know if the prevalence in people correlates with our results observed in the dog population.

Here, dogs were most frequently exposed to Ap compared to the other infectious diseases tested. *Anaplasma* spp. is maintained in the environment by a wide range of hosts such as cattle, wild rodents and cervids. A study from 2013 on Swedish moose (*Alces alces*) found that 100% of the animals tested (n = 234) were seroreactive to Ap antigens [24]. Thus, these animals may also be reservoirr for *Anaplasma* spp. In addition, migratory birds may facilitate the expansion of *Anaplasma-*infected ticks to new regions [25]. Ap has been detected in ticks from Finland and neighbouring countries [26,27]. The prevalence of Ap infection has not yet been studied in Finnish people but studies in Denmark showed a high incidence of human granulocytic ehrlichiosis in humans exposed to ticks [28].

An older study from Denmark found that up to 16.1% of healthy dogs tested were Bb seropositive [29] however, they were not tested for the presence of Ap antibodies. Comparably, a higher prevalence of Ap antibodies (20.7%) was observed in Swedish dogs tested between 1991–1994, in contrast to Bb seroprevalence (4.7%) [30].

The Snap test used in the present study has been described to be highly sensitive and specific [31]. However, serological cross-reactivity between Ap and other related species such as *A. platys*, *E. ewingii* and *E. chaffeensis* have been reported [32]. To date, no infection associated with any of these *Ehrlichia* spp. has been reported in Finland; consequently, it is unlikely that the prevalence observed is based on cross-reactivity. In our study, a single serological testing between September and October, right after the tick season, was performed. For this reason exposure to Ap may have been underestimated. It is possible that some of the dogs in our study were recently exposed or infected with Ap but did not have time to develop detectable antibodies [4,32]. If dogs had been retested a few weeks later, some of them may have seroconverted. The lack of DNA amplification of Ap from dogs that were seropositive could be related to immunological elimination after infection or a low level of infection, and therefore low concentration of DNA in the blood sample. The owners of the dogs in our study were not asked whether their dog had manifested any clinical signs compatible with a tick borne infection in the past, so it is unclear whether the seropositive dogs had been previously infected and eliminated the infection, remaining seropositive.

In the present study, two healthy dogs (one pet dog and one hunting dog, none of which lived in Åland) were infected with Ap, determined by PCR. Although Ap infections are generally associated with acute illness in dogs, subclinical infections have been previously diagnosed in naturally infected dogs [32]. Because it was not possible to perform any follow-up PCR or serology in these two dogs, it remains unknown whether these dogs were able to eliminate the infection without treatment and become PCR negative.

Altogether, 10 client-owned dogs and one hunting dog were seroreactive to Bb antigens. Because the SNAP 4Dx test only detects antibodies as a result of active infection [33], it is possible that the rate of exposure is higher than reported here. Previously, Wilhelmsson *et al*. detected up to 6 different Bb species in ticks that had bitten humans in Åland, which included *B. afzelii, B. garinii, B. valaisiana, B. burgdorferi sensu stricto, B. miyamotoi* and *B. spielmanii*[22]. We did not investigate Bb DNA nor the Bb genospecies in our dogs, thus future studies are necessary to determine the diversity of Bb species in dogs living in Finland.

Based on the high exposure of hunting dogs to ticks and deer keds, which have been found to harbor Bartonellae [18], the authors hypothesized that hunting dogs would be subclinically infected with *Bartonella* spp. However, no dog tested positive for *Bartonella* spp DNA using PCR in the present study. Even though hunting dogs have frequent outdoor access and may be at higher risk of acquiring a vector borne infection, we did not detect a higher prevalence for any tested organism in hunting dogs, compared to the pet dog population. Our data should be cautiously interpreted, because our hunting dog population included hunting dogs that attended dog shows, which may have created a possible bias (the owners of dogs that attend shows may be routinely applying acaricides to their dogs). It is possible that the population of hunting dogs in our study may not be representative of the whole hunting dog population in Finland.

The definitive molecular diagnosis of *Bartonella* infection has proven to be extremely challenging due to the fastidious nature and intracellular tropism of these bacteria for erythrocytes and endothelial cells [16,17,34]. Previously, it was demonstrated that enrichment culture and subculture, followed by PCR amplification, enhances molecular diagnostic sensitivity in dogs [16]. Of the 61 *Bartonella* infected dogs in that study, BAPGM (*Bartonella* alpha-*Proteobacteria* Growth Medium) enrichment culture was required for molecular diagnosis of 36 (59%) dogs [16]. Thus, it is possible that our PCR could have missed some positive cases, but the results indicate anyway a very low prevalence – if any - at the population level. Intravascular infection with *Bartonella* spp. has been associated with a relapsing pattern of bacteremia at 5-day intervals [34]. Consequently, a diagnosis using blood samples collected from a single point of time remains challenging. In fact, obtaining three sequential blood samples during a one-week period may be recommended to increase the sensitivity of the PCR [35]. Optimally, antibody screening against *Bartonella* antigens would have been included in the study. However, serology is diagnostically insensitive. In a previous study, only 25% of *B. henselae* infected dogs and only 50% of the *B. vinsonii berkhoffii* infected dogs were seroreactive by IFA [16].

Finland is situated in the northernmost distribution range of *Ixodes* ticks and therefore the climate change in particular may have a substantial impact on the epidemiology of vector borne infections in this country. Currently, the Åland Islands have adequate temperature conditions for the establishment of *Ixodes* ticks, whereas the temperature in the rest of Finland is not yet optimal for its life cycle. This may explain the geographic differences in seroprevalence observed in our study. If the mean annual temperatures continue to increase, as predicted, it is likely that the population of ticks will continue to expand northward. As a result, the prevalence of seropositive dogs will probably increase gradually in the next decades, which may also correlate with a higher incidence of arthropod borne zoonosis in people.

Several additional factors, together with the climate change, have probably led to the observed emergence of arthropod borne diseases, such as the improvement in the available diagnostic techniques, the development of commercial serological screening tests and an increased awareness among veterinarians and owners about diseases transmitted by arthropods [1]. Outdoor recreation belongs to the Finnish way of life: a large majority of Finns participate in outdoor activities and visit nature during the course of one year. Popular outdoor activities include walking, swimming in natural waters, spending time at a summer cottage, picking berries and mushrooms, biking, hunting, picnicking and collecting wood for household use. These activities increase the risk for people and pet dogs for being bitten by ticks, deer keds and other arthropods [36,37].

## Conclusions

This is the first study to investigate exposure to multiple tick-borne pathogens in dogs in Finland. Our results show that dogs are exposed to at least one of four vector-borne pathogens, including Ap, Bb and Ec. Multivariate logistic regression analysis found a strong association between geographic region (Åland) and being seropositive to Ap and Bb. In addition to Bb and TBEV, Ap infection may be endemic in the Åland Island. Because *I. ricinus* and *I. persulcatus* are capable of transmitting both Ap and Bb to people and small animals, dogs serve as effective sentinel animals to assess the risk of human infection.

## Abbreviations

Ap: *Anaplasma phagocytophilum*; Ec: *Ehrlichia canis*; Bb: *Borrelia burgdorferi*; TBEV: Tick borne encephalitis virus; EDTA: Ethylene diaminetetraacetic acid; IFA: Immunofluorescent antibody assays; OD: Odds ratio; CI: Confidence intervals; BAPGM: *Bartonella* alpha-*Proteobacteria* Growth Medium.

## Competing interests

The authors declare that they have no competing interests.

## Authors’ contributions

CP, SJ, TS and OV designed the study, CP drafted the first version of the manuscript and finalized it. SK collected the blood samples from the hunting dogs. CP and SK collected the data obtained from veterinarians and did some of the DNA extractions. SJ collected the data from blood bank samples. CP, SJ and SK performed the serological tests; CP, SK and KA performed the PCRs. All authors read and approved the final version of the manuscript.
